# Fact Retrieval and Memory Consolidation for a Movement Sequence: Bidirectional Effects of 'Unrelated' Cognitive Tasks on Procedural Memory

**DOI:** 10.1371/journal.pone.0080270

**Published:** 2013-11-07

**Authors:** Rachel Tibi, Zohar Eviatar, Avi Karni

**Affiliations:** 1 Institute of Information Processing and Decision Making and Psychology Department, University of Haifa, Haifa, Israel; 2 Department of Human Biology, the Segol Department of Neurobiology, and E.J. Safra Brain Research Center, University of Haifa, Haifa, Israel; University of Rome, Italy

## Abstract

The generation of long-term memory for motor skills can be modulated by subsequent motor experiences that interfere with the consolidation process. Recent studies suggest that even a non-motor task may adversely affect some aspects of motor sequence memory. Here we show that motor sequence memory can be either enhanced or reduced, by different cognitive tasks. Participants were trained in performing finger movement sequences. Fully explicit instructions about the target sequence were given before practice. The buildup of procedural knowledge was tested at three time-points: immediately before training (T1), after practice (T2), and 24 hours later (T3). Each participant performed the task on two separate occasions; training on a different movement sequence on each occasion. In one condition, interference, participants performed a non-motor task immediately after T2. Half the participants solved simple math problems and half performed a simple semantic judgment task. In the baseline condition there was no additional task. All participants improved significantly between T1 and T2 (within-session gains). In addition, in the baseline condition, performance significantly improved between T2 and T3 (delayed 'off-line' gains). Solving math problems significantly enhanced these delayed gains in motor performance, whereas performing semantic decisions significantly reduced delayed gains compared to baseline. Thus, procedural motor memory consolidation can be either enhanced or inhibited by subsequent cognitive experiences. These effects do not require explicit or implicit new learning. The retrieval of unrelated, non-motor, well established knowledge can modulate procedural memory.

## Introduction

Two behavioral phenomena characterize motor (how to) memory consolidation: i) the emergence of delayed (‘off-line’) gains in performance, which may be sleep dependent, and ii) declining susceptibility to competing experience (interference) (e.g., [1-5]). The nature and the characteristics of an experience that may interact with the process of consolidation are not clear. Interference may occur when the interfering and test tasks share similar kinematic or dynamic features [2,6]. Interference may also be found when both tasks share overlapping neuronal representations but very little kinematic or dynamic features [7]. Recent studies [8,9] suggest that a non-motor declarative encoding task (memory for words) can affect the consolidation phase gains in the implicit learning of a motor sequential task. However, different experiments resulted in apparently conflicting results [8,9]. Nevertheless, these results challenge the classical view of the independence of declarative and procedural memory, and imply an interaction between these two memory systems [10]. 

There is some concern about the findings reported above. In the task most often used in these studies, serial reaction time (SRTT), both declarative and procedural memory systems are involved, but their relative contributions cannot be well controlled [11-13]. Shmuelof and Krakauer [14] have recently pointed out that most studies using the SRTT tap the subject's ability to figure out the required sequence, rather than the generation of 'how-to' motor, procedural knowledge. In addition, there is an untested underlying assumption in the analysis of these experimental data: that the movement sequence is learnt independently of the component movements. The data shown in the Brown and Robertson study (Figure 2 of [8]) for example, clearly show that the training experience affected both the performance of the trained sequence and of the random sequence of movements. Indeed, testing the relationship between component movements and sequence learning in a different finger movement sequence learning task, has revealed that these processes are not independent of each other in the early stages of the learning process [15,16].

**Figure 2:**
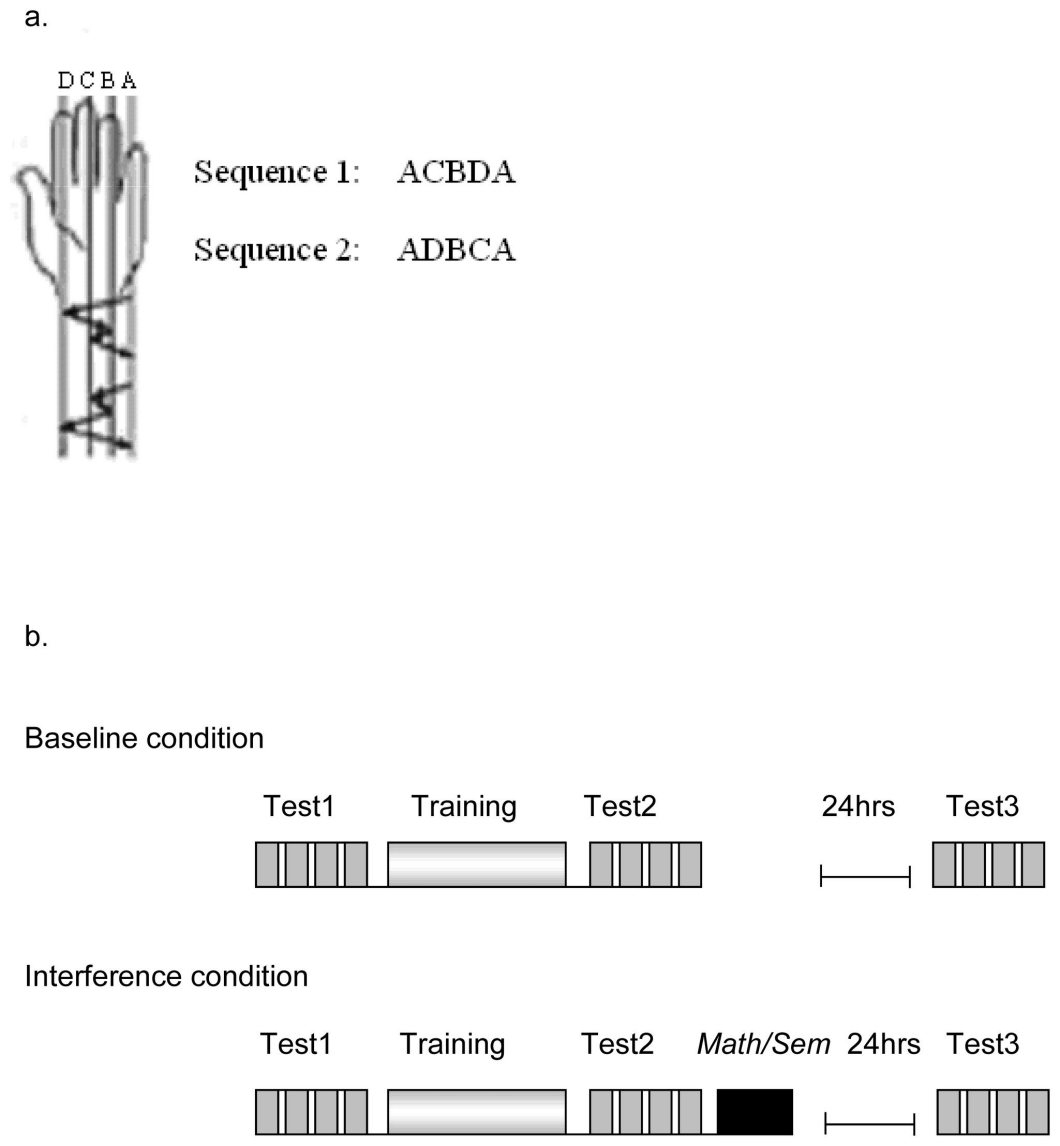
The number of errors and speed in the two conditions across testing times. Error bars are standard errors.

 In the current study we tested the effects of two nonmotor tasks, unrelated to the movement sequence, on the delayed consolidation phase gains in a finger opposition sequence (FOS) task. The task protocol [5,17] ensures that full explicit knowledge of the sequence is available before practice is initiated, so that performance gains reflect the buildup of motor skill (procedural knowledge), and can be analyzed in a empirical manner with no pre-assumptions about types of learning [5,15-18]. 

 Previous studies using the FOS task have shown that the emergence of delayed consolidation phase gains can be effectively blocked, in young adults, by the subsequent performance of different motor tasks, provided that these are introduced within the first few hours after the termination of the FOS training experience [4,5,7,12,15,19,21]. These interference effects were similar to the effects reported by Brashers-Krug et al [2],, who used a manual force-field learning task. 

Ballas et al. [7,21] reported that a hand-writing task introduced immediately after a session of training, interfered with the expression of delayed gains in the FOS task. These results were interpreted as showing that effective interference can occur even when no new learning is required in the interfering task. Rather, it was suggested that the execution/retrieval of motor movement sequences necessary for writing (among highly experienced participants), was the critical interfering element. Importantly, a control task in which Hebrew words were written in Latin orthography (transliterations, requiring different movement sequences) did not affect FOS memory consolidation gains. The model suggested by Brown and Robertson [8,9] posits that the experience of new declarative learning is a critical factor in the interference induced by word-list learning on motor sequence learning [9]. To test this proposal, the two tasks that were studied as potential interference experiences in the current study did not require explicit or implicit new learning. 

One of the tasks was a simple arithmetic task (numbers 1-10). This was chosen because finger individuation and numerical abilities have been linked in both the developmental and acquired forms of Gerstmann's Syndrome (e.g., [20]). A close relationship between the brain representations of finger movement sequences and basic arithmetic (i.e., finger counting) has been proposed (e.g., [22]). The second task was a simple semantic categorization task (living/nonliving) for simple highly frequent concrete nouns. We hypothesized that the math task would result in interference to the expression of delayed gains in the FOS task, whereas the semantic task would not.

## Methods

### Participants

Forty right handed (defined by the Edinburgh questionnaire [23]) University of Haifa undergraduates (15 men) were randomly and equally assigned to two groups. All received course credit for participating. All were native Hebrew speakers, without a history of neurological, psychiatric or hand related orthopedic disorder, and no history of diagnosed learning disabilities or attention deficit. In order to screen out undiagnosed subjects with learning disabilities, all subjects were tested by the Word Recognition One Minute Test (Shatil, E., unpublished, 1996) and the One Minute Arithmetic Test [24]. The study was approved by the University of Haifa Ethics Committee. As the experiment did not include invasive or threatening stimuli, and because participation was voluntary, the University Ethics committee approved obtaining verbal consent from the participants. Participants were informed that they could leave the experiment at any time, with no consequences to them. None chose to do so. Course credit was given to participants who completed the experiment, which comprised the documentation of oral consent.

### Design and Procedure

Each participant took part in two study phases, one week apart, a baseline (non-interference) condition phase and an interference condition phase. The order of the conditions was counter balanced across subjects, with 21 subjects going through the non-interference condition first and a week later, through the interference condition; the rest were given the reverse order of conditions. Both conditions were identical, except that in the interference condition, participants were asked to perform an unrelated cognitive task immediately after the post-training test. One group of 19 subjects performed a semantic decision task (SEM group) and the other group of 21 subjects performed a simple arithmetic problem solving task (Math group).

**Figure 1:**
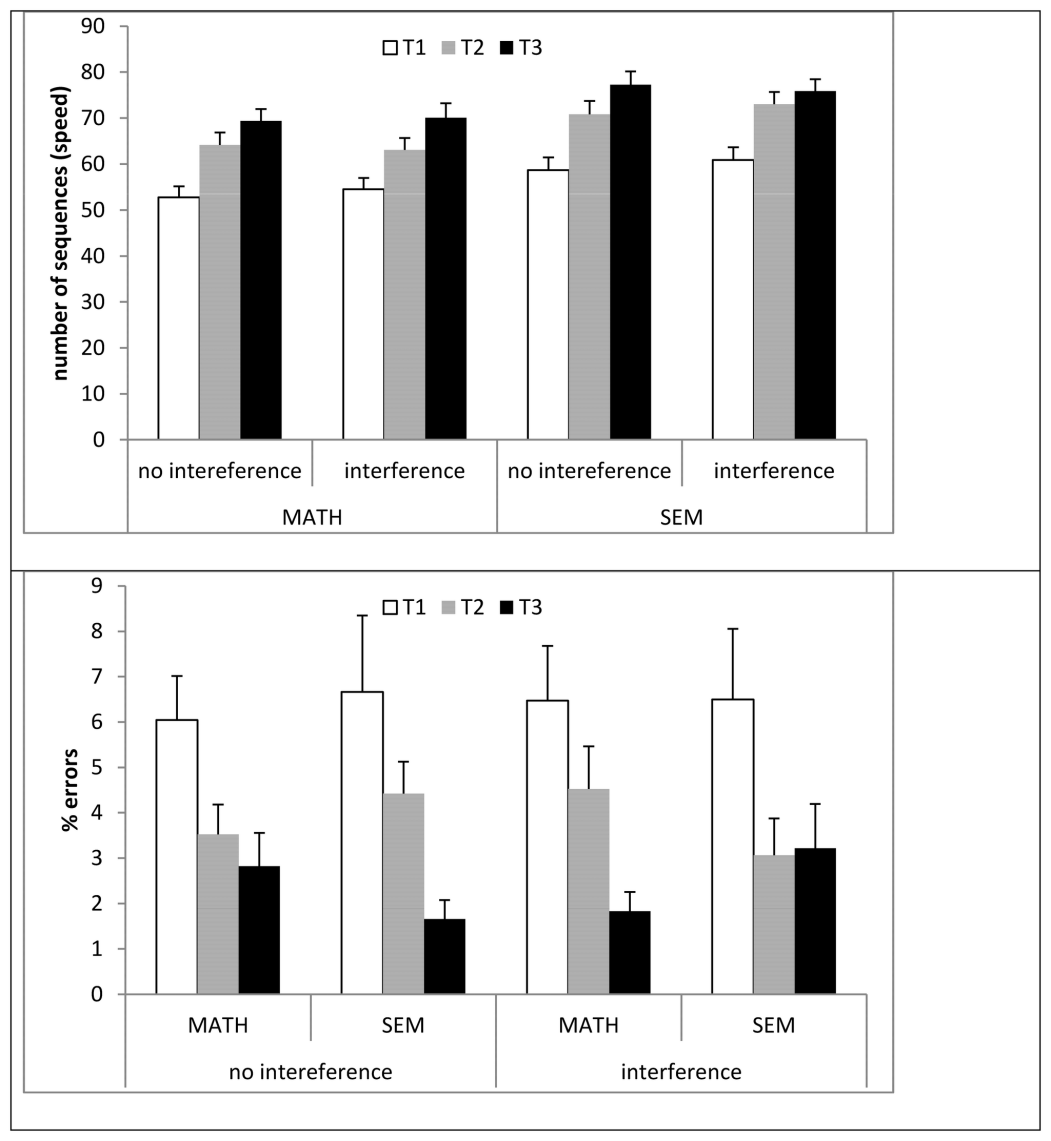
Task and timeline of the experiment. a: The finger-to-thumb opposition task. Subjects were instructed to oppose the finger of the dominant (right) hand to the thumb in one of two possible sequences of five opposition movements (sequences 1,2). One sequence was used in each of the experiment phases (baseline, interference). During instruction, an image of a hand with fingers labeled by letters was presented, while the experimenter said aloud the letters of the specific sequence. Test 1 was initiated only after the participants were able to perform three correct sequences in a row. Training was given in 10 blocks of 16 repetitions of the designated sequence, where each block was preceded by a demonstration of the correct movements. Each trial was cued by an auditory signal. b: The baseline and the interference conditions of the experiment. The conditions were identical except for an interference phase added in the latter condition. Math group solved simple arithmetical exercises and the Sem group made categorical decisions (living/nonliving). In the Tests participants were instructed to perform the sequences as fast and as accurately as possible for an interval of 30s marked by an auditory start and end signal. No feedback was given during testing or training.

 All subjects were trained twice, once in each condition (baseline, interference), in the FOS task using the protocol of Korman et al.[5,15]. The FOS task required participants to learn and practice a 5 element finger-to-thumb opposition movement sequence, as shown in Figure 1. A different sequence was used in each condition (Figure 1). The two conditions were run with an interval of one week between them, which ensured that independent learning occurred for each sequence [7,21]. 

 The participants performed the tasks while sitting on a chair with their left hand positioned on a table at an angle of about 30° from their body, palm perpendicular to the surface of the table, in direct view of a video camera. Participants were instructed to look at a fixation point positioned at a distance of about 1.5 meters at eye level. The task and the required movement sequence were described explicitly: Before the pre-training performance test and beginning practice, the participants were shown an image of a hand with the fingers labeled by Latin letters (A-D), while the experimenter repeatedly named the letters of the specific sequence. The participants reproduced the sequence and were corrected by the experimenter, until they performed it correctly three times in a row. Visual feedback was not given.

In the initial session each participant underwent a pre-training performance test (T1), a training session and an immediate post-training performance test (T2). On the following day, a delayed performance test (T3) was administered. Each training session consisted of 160 repetitions of the sequence, divided into 20 training blocks. During training, the initiation of each sequence was cued by an auditory signal at the rate of 0.4 Hz (2.5 sec per sequence). 

The three performance tests were composed of four 30 sec blocks, initiated by an auditory start signal, during which participants were instructed to continuously and repeatedly tap the sequence until given an auditory stop signal. Participants were explicitly asked to perform the sequence 'as fast and as accurately as possible'. The intervals between blocks were kept constant (30 sec). Participants were instructed not to correct occasional errors and to continue as smoothly as possible. If the stop signal was given during a sequence, they were instructed to complete that sequence. The participants’ performance during the test sessions was video recorded and two measures were extracted from the recordings: speed – number of correct sequences; errors – number of incorrect sequences. 

### Interference Tasks

#### Semantic judgment task (SEM)

 Participants listened to 3 sets of 24 concrete nouns and were asked to say aloud as quickly as possible whether the word represented a living or a nonliving item. Names of tools that involve hand movements were not included in the stimulus list. Voice onset time and accuracy were measured.

#### Arithmetic task (MATH)

The participants listened to 3 sets of 24 arithmetic addition and subtraction problems. All of problems were comprised of single digit numbers, and resulted in answers between 1-10. Participants were asked to provide the answer as quickly and as accurately as possible. Voice onset time and accuracy were measured.

## Results

 The dependent measures were the number of sequences performed (which indexed speed), and the percentage of errors in each test block. 

To test the influence of the order of conditions (performing the non-interference condition first or performing the interference condition first) across both groups, two separate repeated measures general linear model analyses were run for speed and accuracy as dependent variables, with condition (baseline; interference) and test (2 levels: T1, T2 for within session analysis; T2, T3 for between session analysis) as within subject variables and order (first baseline; first interference) and interference group (Math group, Sem group) as between subject variables. No significant main effects of order were found for speed in the within-session phase (F = 1.086; p = 0.304) or in the between-session phase (F = 1.213; p = 0.278), and no significant order effects were found for accuracy (F = 0.249; p = 0.625; and F = 0.141; p = 0.709; within and between session, respectively). Moreover, no significant order x group interactions were found for speed (F = 0.187; p = 0.668; F = 0.035; p = 0.852; within and between session, respectively), and similarly, no significant order x group interactions were found for accuracy (F = 0.37; p = 0.547; F = 0.268; p = 0.608; within and between session, respectively). Therefore, order was pooled in all of the following analyses. 

Analyses of Variance (ANOVA) were computed with Condition (baseline, interference), interference group (MATH, SEM) and Test (T1, T2, & T3) as independent factors, for each dependent measure. The overall analyses revealed somewhat different effects for speed and for errors.

For speed, only the main effects of Test (F(2,76)=306.52,p<.0001) and of interference group (F(1,38)=4.67, p<.05) were significant. It can be seen in Figure 2, and planned comparisons confirmed, that performance was significantly faster in T2 than in T1 (F1,38)=269.11,p<.0001) and faster in T3 than in T2 (F(1,38)= 105.81,p<.0001). Thus, overall, we see both *within-session learning* (the difference between T1 and T2), and *delayed gains* (the difference between T2 and T3). In addition, the SEM group was faster than the MATH group in all of the tests, although this difference was not significant at T1 (MATH=52.7; SEM=58.8, p>.10), was marginal at T2 (MATH=64.1; SEM=70.8, p>.08), and significant at T3 (MATH=69.4; SEM=77.3, p<.05). Examination of the interaction between interference group and time of testing showed no significant interaction in the baseline, no-interference condition (p>.56), and a trend towards a significant interaction in the interference condition (p=.10). This is examined in more detail below.

For percent errors, the 3-way interaction between condition, interference group, and time of test was marginal, F(2,76)=2.71, p=.07 The only other significant effect was of time of test, F(2,76)=26.12, p<.0001. The interaction is shown in the bottom panel of Figure 2. Separate analyses of the baseline and the interference conditions revealed that the interaction between interference group and time of testing was not significant in the baseline condition (p>.11) and was significant in the interference condition: F(2,76)=3.14, p<.05). It can be seen that there are opposing effects of the differences between T2 and T3 in the interference condition between the MATH and SEM group. In the MATH group, there are less errors in T3 (1.8%) than in T2 (4.5%) (delayed gains), and this difference is significant, F(1,20)=17.19,p<.005. In the SEM group, there are more errors at T3 (3.2%) than at T2 (3.06%) in the interference condition (no delayed gains), but this difference is not significant, p>.8. In order to make sure that the interaction does not result from random differences between the groups, we tested the simple main effect of group in each condition at each testing time. The groups did not differ from each other at any of the testing times (p>.15) 

**Figure 3:**
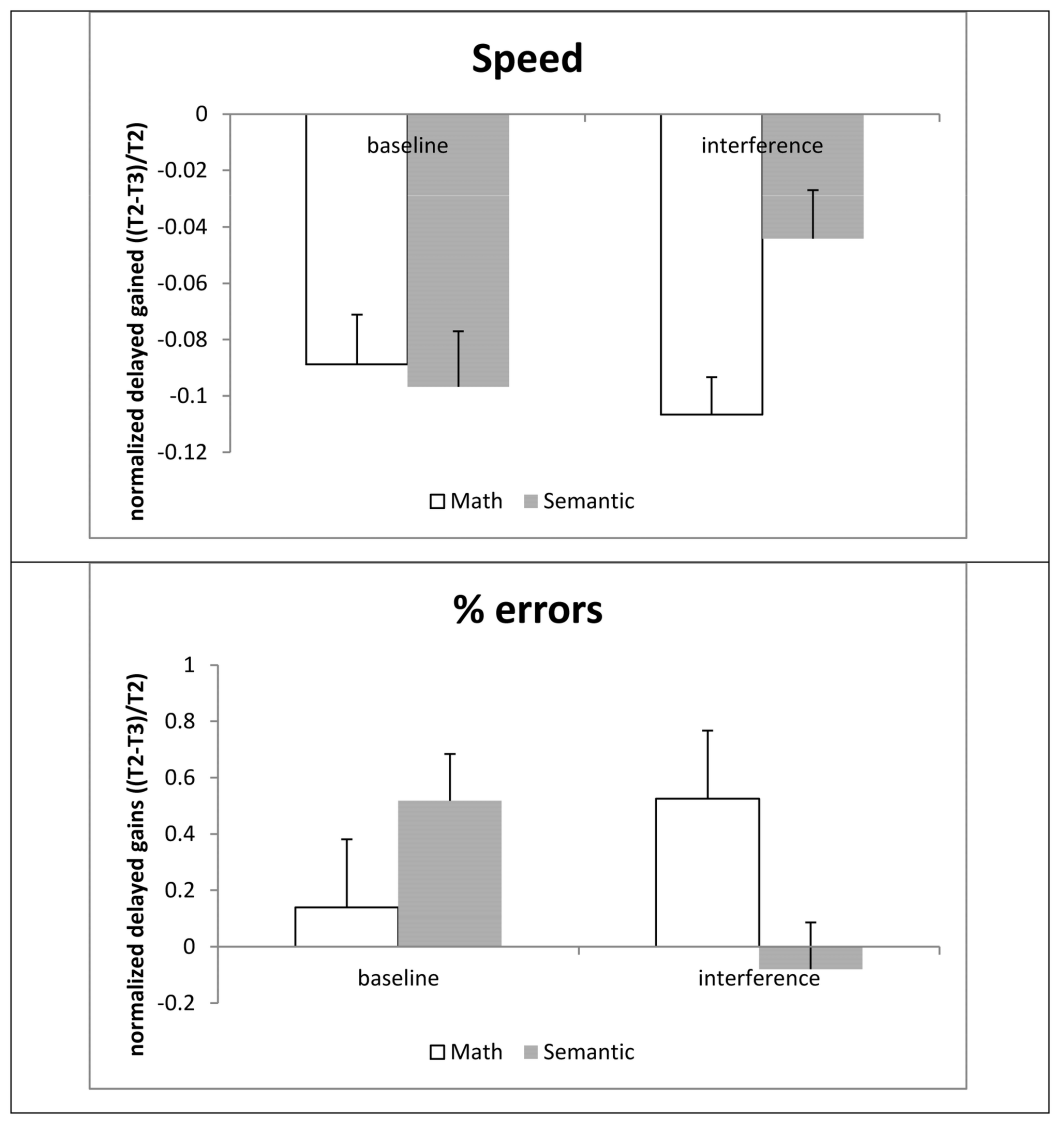
Opposing effects of the two tasks on delayed gains. Delayed gains were computed as the difference in errors and speed between T3 and T2, and then normalized for the difference in T2, the delayed gains=(T3-T2)/T2. Note that for errors, a negative value indicates a gain in accuracy, whereas for speed, a larger value indicates more sequences performed within the test interval.

 In order to specifically examine the differential effects of the two interference conditions on the expression of *delayed gains*, and in order to control for the effects of the difference between the groups in T2 in the interference condition, we computed a normalized score for the delayed gains, by dividing the difference between T2 and T3 by T2. In order to avoid division by 0, when T2 =0 in the % error scores, it was changed to 1. A positive number in error scores and a negative number in speed scores indicate delayed gains (less errors and more correct taps performed in T3 than in T2) (Figure 3). An analysis of these scores revealed a significant interaction between condition and interference group in both measures (speed: F(1,38)=4.32, p<.05, % errors: F(1,38)=7.80, p<.005), and no main effects (p>.09 or larger). In order to check that this normalization equalizes performance at baseline, we compared the normalized delayed gains separately in each condition. In the baseline condition, normalized delayed gains were not significantly different in the two groups (speed: Math=-.089, Sem=-.096, p>.7 ; errors: Math=.138 Sem=.52, p>.2). In the interference condition, the difference in normalized delayed gains in the two groups was significant in both measures (speed: Math=-.11, Sem=-.04, F(1,38)=8.93,p<.005; errors: Math=.52. Sem=-0. 08, F(1,38)=6.95, p<.05). Overall, the improvement in speed was accompanied by a significant reduction in the number of errors committed (i.e., there was no speed-accuracy tradeoff). As seen in Figure 3, the MATH task resulted in a facilitation of delayed gains, both in terms of number of sequences tapped and in terms of the errors committed, compared to baseline. On the other hand, the SEM task resulted in interference effects in both measures, compared to baseline. 

## Discussion

The unexpected aspect of the current results is that one apparently unrelated non-motor task, performed after the termination of the training experience, can effectively enhance motor memory consolidation, while another apparently unrelated non-motor task, can interfere with motor memory consolidation. Overall, these results constitute a challenge to the view that interference (or enhancement) of memory consolidation must necessarily be ascribed to a competition (or cooperation) in neuronal resources shared between the trained and the subsequent tasks. It has been proposed [2,6,21,25–27] that interference may occur when both initial training and subsequent experience activate overlapping neural representations in specific brain areas. The need to adapt to the demands of the second task may eliminate or supersede the settings of the initial task with an advantage for the most recent experience For example, in line with the synaptic-tagging hypothesis (e.g., [28]), Balas et al. [21] proposed that when the initial and subsequent experiences activate different groups of synapses at a putative shared neuronal level, e.g., within the population of neurons active in the performance of both tasks, there are possible grounds for competition. Thus, memory consolidation processes triggered by the activation of a subset of synapses subserving the initial task may be taken over by the activation of a different subset of synapses subserving the subsequent (interference) experience. The modification of the former group of synapses, representing the long-term memory of the initial task, may be slowed or left incomplete [28,29]. A similar line of thought suggests that if the two experiences (initial learning and subsequent experience) activate the same set of synapses or contribute together to the consolidation process, there are possible grounds for enhancement. Alternatively, as proposed by Albouy et al. [10] and Brown and Robertson [8,9], enhancement or interference reflect indirect effects, that is, interactions between two systems, rather than shared neuronal resources. It may be the case, that the subsequent experience indirectly affects the ongoing memory consolidation process by recruiting or inhibiting a competing memory consolidation system (the declarative memory system). However, the current results indicate that competition between consolidation processes in two memory systems may not be the crucial factor, because neither the MATH nor the SEM conditions required novel memory consolidation. Both tasks required retrieval from well established long-term memory and possible reconsolidation [30], but nevertheless, resulted in opposite effects. A model that would predict these divergent effects is not available currently. 

One possibility is that the facilitating effect of the MATH task may have resulted from a positive relationship between numeric processing and finger individuation. Habits of finger counting have been proposed as an explanation for cultural differences in number line representation ( e.g., [22]). Finger individuation and numerical abilities have previously been linked in both the developmental and acquired forms of Gerstmann's Syndrome (e.g., [20,31]) although the question of whether the respective representations in parietal cortex, overlap or not, is still under debate [32]. Parietal cortex representations have been implicated in the representation of newly trained movement sequences [33,34], providing a possible neural substrate shared with arithmetic problem solving. 

It is difficult to account for the interference effects of the semantic decision task. Moreover, previous studies have reported both enhancing [8] and inhibiting [9] effects of a wordlist memorizing and free recall task. These authors ascribed both results to indirect effects: reciprocal interactions between the declarative and procedural memory systems. An alternative mechanism in line with the standard model of 'interference as competition for shared neural resources' can be considered. For example, a recent study indicated a role for dorsolateral pre-frontal cortex (DLPFC) in the immediate post-training consolidation phase [35] for a movement sequence; word list memorization and immediate recall are known to enlist these brain regions (e.g., [36]). In addition, parietal regions have also been shown to contribute to word retrieval ( e.g., [37]) providing further substrates for competition. 

Our data converge with behavioral and neuroimaging data (e.g., [12]) that suggest a less compartmentalized view of the dichotomy between the two dissociated memory systems: declarative knowledge which is associated with explicit learning, and procedural knowledge which is associated with implicit learning. The current data do not support the notion that delayed gains related to procedural memory consolidation can be better expressed if declarative memory is engaged by another task during the consolidation phase [8,11]. Rather, our results suggest that it is the specific content of the subsequent experience which may be crucial.

Our results show that subsequent experience can affect skill consolidation processes in both directions: interference, but also facilitation. Clarifying the interrelationships between cognitive-nonmotor and motor tasks at the level of memory mechanisms for skill (how-to knowledge) could have theoretical as well as practical implications for experimental procedures, for rehabilitation, and in education. The effectiveness of motor skill memory may depend not only on the order and time interval between tasks, but also on the nature of presumably 'unrelated' tasks. 
